# Quantification of the whole-body burden of radiographic osteoarthritis using factor analysis

**DOI:** 10.1186/ar3501

**Published:** 2011-10-25

**Authors:** Amanda E Nelson, Robert F DeVellis, Jordan B Renner, Todd A Schwartz, Philip G Conaghan, Virginia B Kraus, Joanne M Jordan

**Affiliations:** 1 Thurston Arthritis Research Center, University of North Carolina, 3300 Thurston Building CB 7280, Chapel Hill, NC, 27599, USA; 2 Department of Health Behavior and Health Education, Gillings School of Global Public Health, University of North Carolina, 302 Rosenau Hall, Chapel Hill, NC, 27599, USA; 3 Department of Radiology, University of North Carolina, 101 Manning Drive, Chapel Hill, NC, 27514, USA; 4 Department of Biostatistics, Gillings School of Global Public Health, University of North Carolina, 3106E McGavran-Greenberg Hall, Chapel Hill, NC, 27599, USA; 5 Section of Musculoskeletal Disease, University of Leeds & NIHR Leeds Musculoskeletal Biomedical Research Unit, Chapel Allerton Hospital, Chapeltown Road, Leeds, LS7 4SA, UK; 6 Department of Medicine, Duke University Medical Center, 595 La Salle St, Durham, NC, 27710, USA

**Keywords:** Radiography, osteoarthritis, factor analysis

## Abstract

**Introduction:**

Although osteoarthritis (OA) commonly involves multiple joints, no widely accepted method for quantifying whole-body OA burden exists. Therefore, our aim was to apply factor analytic methods to radiographic OA (rOA) grades across multiple joint sites, representing both presence and severity, to quantify the burden of rOA.

**Methods:**

We used cross-sectional data from the Johnston County Osteoarthritis Project. The sample (*n *= 2092) had a mean age of 65 ± 11 years, body mass index (BMI) 31 ± 7 kg/m^2^, with 33% men and 34% African Americans. A single expert reader (intra-rater κ = 0.89) provided radiographic grades based on standard atlases for the hands (30 joints, including bilateral distal and proximal interphalangeal [IP], thumb IP, metacarpophalangeal [MCP] and carpometacarpal [CMC] joints), knees (patellofemoral and tibiofemoral, 4 joints), hips (2 joints), and spine (5 levels [L1/2 to L5/S1]). All grades were entered into an exploratory common factor analysis as continuous variables. Stratified factor analyses were used to look for differences by gender, race, age, and cohort subgroups.

**Results:**

Four factors were identified as follows: IP/CMC factor (20 joints), MCP factor (8 joints), Knee factor (4 joints), Spine factor (5 levels). These factors had high internal consistency reliability (Cronbach's α range 0.80 to 0.95), were not collapsible into a single factor, and had moderate between-factor correlations (Pearson correlation coefficient r = 0.24 to 0.44). There were no major differences in factor structure when stratified by subgroup.

**Conclusions:**

The 4 factors obtained in this analysis indicate that the variables contained within each factor share an underlying cause, but the 4 factors are distinct, suggesting that combining these joint sites into one overall measure is not appropriate. Using such factors to reflect multi-joint rOA in statistical models can reduce the number of variables needed and increase precision.

## Introduction

Generalized osteoarthritis (OA) as a disease entity has been described for well over 100 years [1]. In 1952, Kellgren described multiple joint OA involvement (feet, facet joints, knees, hips, and other limb joints) among individuals with Heberden's nodes and/or carpometacarpal (CMC) OA [2]. He states in this paper that although many individuals had "polyarticular OA" without nodes or CMC OA, "we are not yet prepared to make this diagnosis [primary generalized OA] in the absence of Heberden's nodes or arthritis of the first CMC joints [2]." Since that time, published reports have defined generalized OA in a variety of ways, including nodal with large joint OA, more than three or five joints or joint sites involved [3], summed numbers or grades of affected joints [4-7], multiple hand joints [8], or nodal hand OA with other joints involved [9]. However, there remains no widely accepted and universally used definition of generalized OA in the literature despite widespread use of the term itself.

This lack of an accepted definition makes it difficult to quantify the effect of multiple joint involvement on OA outcomes, leading to the common practice of focusing on a single joint site without considering the contribution of other involved sites. This is particularly problematic in the setting of systemic factors, such as functional disability, performance-based outcomes requiring the use of multiple joint sites, serum/urine biomarkers, and genetics, which are necessarily a reflection of the whole-body burden of OA and the impact of that burden on the individual. In this setting, it would be advantageous to have a parsimonious composite measure(s) that could be included in a statistical model to account for the whole-body burden of OA.

Factor analysis, a method used for 80 years in the social sciences [10-12], provides a way to determine whether a set of variables has one or more relatively global underlying variables that can account for the observed correlations among the analyzed items [10]. Thus, this analytic approach can clarify the extent to which one or more explanatory concepts or dimensions account for most of the shared variation among the variables. Composite scores combining such variables can then be used in further statistical modeling of an outcome of interest, reducing dimensionality of models and increasing estimate precision. Factor analysis was developed for intelligence testing [11], then expanded to other psychological variables, and now frequently used in validation of multi-item questionnaires in a variety of disciplines [13-16]. Factor analysis and related psychometric methods have been used to evaluate multiple clinical instruments used in arthritis, including the Arthritis Impact Measurement Scales Health Status Questionnaire (AIMS2) [17], Western Ontario and McMaster Universities Arthritis Index (WOMAC) [18], and Australian/Canadian Osteoarthritis Hand Index (AUSCAN) [19].

To our knowledge, these methods have only twice been applied to radiographic data, and then only for the hand [20,21]. However, the potential strength of factor analysis in understanding the whole body burden of radiographic OA (rOA) lies in its ability to account for presence and severity of rOA in multiple joint sites by including the full range of all of the individual radiographic scores. We were therefore interested in applying factor analytic methods to Kellgren-Lawrence global (KL, 0 to 4) and Burnett atlas joint features (0 to 3) radiographic grades [22,23] across multiple joint sites as a way to formulate composite scores of multi-joint rOA, encompassing both presence and severity, using data from participants in the Johnston County Osteoarthritis Project (*JoCo OA*).

## Materials and methods

The analysis used data from the *JoCo OA*, a population-based prospective cohort study of non-institutionalized African American and white men and women, living in rural North Carolina, aged 45 years and older, both with and without OA, which has been described previously [24]. All participants signed informed consents, and completed two home interviews and one clinic visit with physical examination, including functional measures and radiographs, administered by trained study personnel. Multi-joint radiographs were added at the cohort enrichment (2003 to 2004) and second follow up (2006 to 2010), so data from these time points were used for the current analysis (total *n *= 2121). Data from a given individual was included from only one time point.

Self-reported age, gender, and race were obtained from interviewer-administered questionnaires, while body mass index (BMI) was calculated in kg/m^2 ^from height (cm) and weight (kg) measured during clinic examination by trained study examiners. This cross-sectional analysis included demographic, clinical, and radiographic data collected at the same time for each participant (either during the 2003 to 2004 or 2006 to 2010 time period). The *JoCo OA *has been continuously approved by the Institutional Review Boards of the University of North Carolina and of the Centers for Disease Control and Prevention in Atlanta, GA.

### Radiographs

**Figure 1:**
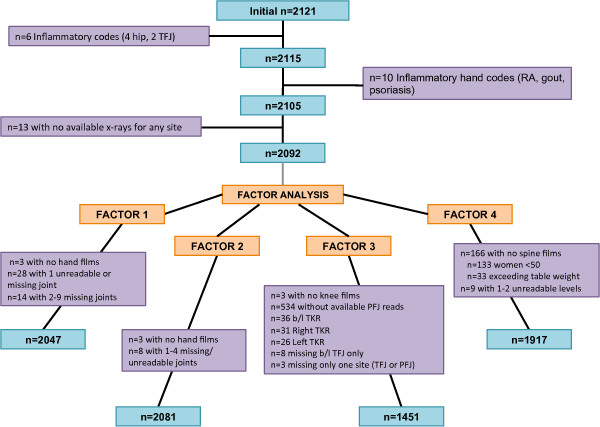
**Participant inclusion and exclusion**. Flow diagram showing sample size and exclusions for the analyses. Individuals with evidence of inflammatory changes were excluded, as were those missing radiographic data for each factor; the reasons for missing data are detailed.

Posteroanterior radiographs of the bilateral hands were read for KL grade [22] at each of 30 joints (distal interphalangeal (DIP), proximal interphalangeal (PIP), metacarpophalangeal (MCP), CMC, thumb IP and MCP). Fixed flexion, weight-bearing posteroanterior views of the tibiofemoral joint (TFJ) using the Synaflexer™device (CCBR-Synarc, San Francisco, CA, USA) were read for KL grade. Sunrise views of the patellofemoral joints (PFJ) were read for osteophytes (OST) [25] using the Burnett atlas [23]. PFJ films were added later in the study and had not all been read at the time of this analysis (see also Figure 1). Anteroposterior supine pelvis films were used to assess KL grade at the hips; these films were not performed in women under age 50 years. Lateral lumbosacral spine (LS) films (taken with the participant lying on his/her left side) were read for OST and disc narrowing (DN) at five levels (L1/2 through L5/S1). Joints that had undergone replacement were not included, because no KL or OARSI score could be assigned. Individuals with radiographs suggestive of an underlying inflammatory condition were excluded. All films were read by a single experienced musculoskeletal radiologist (JBR) previously shown to have high intra- and inter-rater reliability (κ = 0.89 and 0.86, respectively) [26].

Prior to factor analysis, *a priori *definitions for rOA were determined, such that 1) joints that were not included in any factor could still be included in a model to allow estimates to reflect the whole body burden of rOA and 2) sample characteristics for OA could be summarized. For the hand joints, TFJ, and hip, a KL grade of 2 or more was considered to be diagnostic of rOA in any given joint. For the PFJ, any OST of 2 or more was considered to indicate PFJ OA. LS OA was defined if both OST and DN graded 1 or more were present at a single level [9].

### Factor analysis

In this paper, the term factor analysis refers to an exploratory common factor analysis, in which the factors represent hypothetical (latent) variables that are being estimated. Determination of the number of factors to retain can be performed utilizing eigenvalues, scree plots, factor interpretability, and/or parallel analysis, although there remains a level of subjectivity to this decision [10]. We used a scree plot, which allows visual comparison of relative eigenvalues [27]. Variables that did not load well (≤ 0.4) or had cross-loadings, indicating weak relations to the latent variable, were dropped. An oblique rotation, which allows the factors to correlate with each other, was then applied. Higher order factor analysis was used to determine whether the identified factors represented a single latent variable (and could therefore be combined) or contained independent information that would be lost through combination. Cronbach's alpha statistic was calculated as a measure of internal consistency reliability for all factors. Separate factor analyses were performed for each gender (men and women), race (African American and white), age (< 65 compared with 65+ years) and cohort (cohort enrichment versus second follow-up cohorts) subgroup; qualitative comparisons of factor structure and Cronbach's alpha were performed. All analyses were performed in Stata 11.0 (StataCorp, College Station, TX, USA).

## Results

### Sample characteristics

The inclusion of subjects in the current analysis is presented, along with reasons for exclusion, in Figure 1. Sixteen individuals were excluded due to evidence of inflammatory disease on radiographs; the remainder of excluded individuals were missing radiographic data as shown. Detailed sample characteristics for the total sample (*n *= 2,092) and the subset with interpreted radiographs for all joint sites (*n *= 1,373) are shown in Table 1. The subset with all radiographs was slightly older (67.3 versus 65.1 years), with a similar mean BMI and proportion of men and African Americans. About 45% of the participants had OA of the DIP joints, 30% had PIP OA, and 9% had MCP OA. Slightly under one third had CMC OA, while slightly over one third had hip OA. Forty percent had TFJ OA, about 10% had any PFJ OST grade 2 or more, and 60% had LS OA by our definition, with the subset overall similar to the total population (Table 1).

**Table 1:** Sample characteristics

	Total sample (*n *= 2,092)*	Subset with all radiographs (*n *= 1,373)
	*Mean (SD) or n (%)*	*Mean (SD) or %*
**Age (years)**	65.1 (10.9)	67.3 (9.7)
**BMI (kg/m^2^)**	31.3 (7.1)	30.8 (6.4)
**Male**	695 (33.2)	472 (34.4)
**African American**	709 (33.9)	438 (31.9)
**DIP OA^†^**	902 (43.2)	637 (46.4)
**PIP OA**	595 (28.5)	419 (30.5)
**MCP OA**	195 (9.4)	132 (9.6)
**CMC1 OA**	577 (27.7)	416 (30.3)
**TFJ OA**	822 (39.8)	554 (40.4)
**PFJ Ost > = 2**	174 (11.8)	142 (10.3)
**Hip OA**	643 (33.8)	475 (34.9)^§^
**LS OA^‡^**	1,138 (59.2)	836 (60.9)

*For total sample (at least one radiograph, see Figure), For LS OA *n *= 1,923; 2,068 for TFJ OA; 1,901 for Hip OA; 1,481 for PFJ Ost; 2,088 for DIP and PIP OA; 2,086 for MCP and CMC OA

^†^Where OA is defined as KL grade > = 2 at any DIP, PIP, CMC, MCP, TFJ, or hip joint

^‡^Defined as having at least one lumbar level (L1/2 to L5/S1) with both ost and DN OARSI grade > = 1

^§^Hip OA *n *= 1,360

BMI, body mass index; CMC, carpometacarpal joint; DIP, distal interphalangeal joint; LS, lumbosacral spine; MCP, metacarpophalangeal joint; OA, osteoarthritis; OST, osteophytes; PFJ, patellofemoral joint; PIP, proximal interphalangeal joint; SD, standard deviation; TFJ, tibiofemoral joint.

### Factor analysis

**Figure 2:**
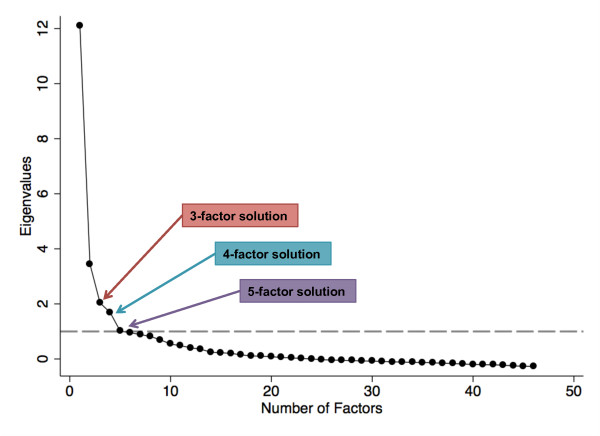
**Scree plot**. Scree plot using Johnston County Osteoarthritis Project radiographic data. Based on the eigenvalue (the dashed line indicates an eigenvalue of 1.0) and scree plot criteria, a three-, four-, or five-factor solution would be acceptable. Subjective examination based on interpretability and simple structure led the authors to use a four-factor solution.

All radiographic variables (KL or Burnett atlas joint features scores) were entered into a factor analysis simultaneously as continuous scores, such that each joint site had a score of 0 to 4 or 0 to 3. We explored three-, four-, and five-factor solutions, as all of these were reasonable based on eigenvalues above 1 and scree plot findings (Figure 2). Balancing interpretability and simple structure, we selected a four-factor solution. Then, variables with weak or cross-loadings were dropped from the analysis. The hips had loadings of less than 0.2 in all solutions and were dropped. The first MCPs also had poor loadings (< 0.4), as well as cross-loadings, and were dropped. In a three-factor solution, the separate LS variables (first the DN grades, then the OST grades) had low loadings and so these were dropped, as were the CMC variables (loadings < 0.4). However, when all LS variables were simultaneously included and a four-factor solution was used, these variables had acceptable loadings, as did the CMC joint, and all were retained in the present four-factor solution. In contrast, a five-factor solution caused the LS variables to cross-load onto factors 4 and 5 with lower loadings overall. An oblique rotation was then applied, as we deemed it unlikely that radiographic variables at different joint sites would be entirely independent. In this final solution, we identified four factors that can be thought of as latent variables underlying the individual radiographic variables contained in each (Table 2). The four factors were moderately correlated, with Pearson correlation coefficients ranging from 0.24 to 0.40 (Table 3).

**Table 2:** Factor loadings* for each radiographic variable in the four-factor solution after oblique rotation

rOA Variable	Factor			
	IP/CMC	MCP	Knee	Spine
L 1^st ^IP	0.512			
R 1^st ^IP	0.550			
L CMC 1	0.442			
R CMC 1	0.429			
L 2^nd ^DIP	0.833			
L 3^rd ^DIP	0.840			
L 4^th ^DIP	0.814			
L 5^th ^DIP	0.786			
R 2^nd ^DIP	0.796			
R 3^rd ^DIP	0.797			
R 4^th ^DIP	0.815			
R 5^th ^DIP	0.786			
L 2^nd ^PIP	0.682			
L 3^rd ^PIP	0.707			
L 4^th ^PIP	0.711			
L 5^th ^PIP	0.669			
R 2^nd ^PIP	0.685			
R 3^rd ^PIP	0.694			
R 4^th ^PIP	0.749			
R 5^th ^PIP	0.636			
L 2^nd ^MCP		0.595		
L 3^rd ^MCP		0.649		
L 4^th ^MCP		0.614		
L 5^th ^MCP		0.605		
R 2^nd ^MCP		0.551		
R 3^rd ^MCP		0.589		
R 4^th ^MCP		0.671		
R 5^th ^MCP		0.602		
Right TFJ			0.699	
Left TFJ			0.746	
Right PFJ avg*^†^*			0.829	
Left PFJ avg*^†^*			0.856	
L1/2 DN				0.555
L2/3 DN				0.697
L3/4 DN				0.622
L4/5 DN				0.474
L5/S1 DN				0.427
L1/2 OST				0.560
L2/3 OST				0.652
L3/4 OST				0.568
L4/5 OST				0.416
L5/S1 OST				0.384

*Only loadings > 0.2 are shown

**^†^**PFJ presented for simplicity as average osteophytes across four surfaces (lateral femoral, lateral patellar, medial femoral, medial patellar), nearly identical loadings are obtained for individual surface scores.

CMC, carpometacarpal joint; DIP, distal interphalangeal joint; DN, disc narrowing; IP, interphalangeal joint; MCP, metacarpophalangeal joint; OST, osteophytes; PFJ, patellofemoral joint; PIP, proximal interphalangeal joint; rOA, radiographic osteoarthritis; TFJ, tibiofemoral joint.

**Table 3:** Pearson correlation coefficients between factors in the rotated four-factor solution

	IP/CMC factor	MCP factor	Knee factor	Spine factor
IP/CMC factor*	1.0			
**MCP factor**	0.44	1.0		
**Knee factor**	0.30	0.36	1.0	
**Spine factor**	0.24	0.30	0.40	1.0

*IP/CMC factor: Thumb IP, DIP 2-5, PIP 2-5, CMC1; MCP factor: MCP 2-5, Knee factor: TFJ and PFJ; Spine factor: Disc space narrowing and osteophytes L1/2 to L5/S1.

IP, interphalangeal joint; MCP, metacarpophalangeal joint; PFJ, patellofemoral joint; PIP, proximal interphalangeal joint; TFJ, tibiofemoral joint.

The first can be defined as an IP/CMC Factor, and contains the thumb IP and CMC, DIPs 2 to 5 and PIPs 2 to 5 of both hands (factor loadings 0.43 to 0.84). The lowest loadings on this factor were observed for the CMC (0.43 to 0.44), compared with 0.51 to 0.84 for the other included joints. The second factor is an MCP Factor, and includes the MCP joints of the second to fifth fingers of both hands (loadings 0.55 to 0.67). The third is the Knee factor, including the TFJ and PFJ of both knees (loadings 0.70 to 0.86). The fourth factor is the spine factor including DN and OST variables from the five LS levels L1/2 to L5/S1 (loadings 0.38 to 0.70). The lowest loadings were observed for DN and OST at the L4/5 and L5/S1 levels (0.38 to 0.47) compared with the other levels (0.55 to 0.70), but for interpretability, all LS levels were retained. Cronbach's alpha statistic was calculated for each of the final factors to give a measure of reliability, and ranged from 0.80 for the spine factor to 0.95 for the IP/CMC factor (Table 4). The results were essentially unchanged when stratified by gender (α = 0.77 to 0.96), race (α = 0.78 to 0.96), age (α = 0.74 to 0.95), or cohort (α = 0.77 to 0.95) subgroups.

**Table 4:** Cronbach's alpha (internal consistency reliability) and average inter-variable correlation for the 4 factors

Factor*	n (obs)^†^	n (variables)^‡^	Cronbach's alpha	Average inter-variable correlation
**IP/CMC**	2047	20	0.95	0.51
**MCP**	2081	8	0.81	0.35
**Knee**	1451	4	0.85	0.59
**Spine**	1917	10	0.80	0.29

*IP/CMC factor: Thumb IP, DIP 2-5, PIP 2-5, CMC1; MCP factor: MCP 2-5, Knee factor: TFJ and PFJ; Spine factor: Disc space narrowing and osteophytes L1/2 to L5/S1.

**^†^**Number of observations with data for a factor

^‡^Number of radiographic variables included in a factor

CMC, carpometacarpal joint; DIP, distal interphalangeal joint; IP, interphalangeal joint; MCP, metacarpophalangeal joint; PFJ, patellofemoral joint; PIP, proximal interphalangeal joint; TFJ, tibiofemoral joint.

We were interested in the possibility of a single, higher order factor that could be defined as a "generalized rOA" score. However, in our analysis, higher order factor analysis of the four factors showed that they should not be combined into a single factor. For comparison, the individual factors had eigenvalues ranging from 11.7 (IP/CMC factor) to 1.7 (spine factor), compared with values not exceeding 1.1 for the higher order analysis. The loadings of the four factors on the higher order factor ranged from 0.47 to 0.56. In addition, the reliability for this single higher order factor was lower than any of the individual factors at 0.65.

## Discussion

We have employed factor analysis as a way of understanding the latent factors underlying radiographic variables in OA. We report a four-factor solution as follows: 1) IP/CMC factor (20 joints), 2) MCP factor (eight joints), 3) knee factor (four joints), and 4) spine factor including DN and OST (5 levels, 10 variables). Use of a composite (e.g., an average or a standardized score) for each of these four factors in a multiple regression model would result in representation of all 20 + 8 + 4 + 10 = 42 underlying variables (or 37 distinct joint sites) through only four explanatory variables. Taking the MCP factor as an example, the individual scores from the eight MCP joints could be added and divided by eight, resulting in a single, average value for the MCP factor rather than eight individual scores. This allows consideration of the whole body burden of rOA in a parsimonious model, resulting in reduced dimensionality and increased precision of the resultant estimates. This is particularly important when considering a systemic outcome, such as a molecular or genetic biomarker, or a performance or disability score, which is likely influenced by the overall rOA burden and not only by the specific joint of interest in a study. The results were similar by age, between men and women, between African Americans and whites, and between members of the two cohorts.

Factor analysis involves some subjective decision-making, particularly when selecting the number of factors to retain and dropping variables that do not load well. The benefits of the four-factor solution are inclusion of the LS and CMC variables, which were not part of the three-factor solution. However, the spine factor has the lowest reliability and contains one variable (osteophytes at L5/S1) with loading less than 0.4, and the reliability of the knee factor is slightly reduced in the four-factor (α = 0.85) compared with the three-factor (α = 0.87) solution. The elimination of the hip variables was robust and similar across all solutions, however, supporting the idea that the hip is not part of the generalized OA construct. Also dropping out in all solutions were the first MCP variables, for reasons that are not immediately obvious, as these joints were involved at a similar frequency to the other MCP joints.

Our results do not support a single underlying latent variable for all joint sites, and are therefore not consistent with the concept of "generalized OA." This is in agreement with a study by MacGregor, et al, who used structural equation modeling to assess relations between knee, hip, PIP, DIP, and CMC rOA (also using the KL grading system) in a population of female twins accounting for shared genetics [28]. The authors found that, while each individual joint site demonstrated genetic influence (heritability estimates 28 to 68%), there was a moderate genetic correlation only between DIP and PIP joints, and little or no correlation between other joint sites [28]. MacGregor et al, conclude from their study that there was "little evidence of the phenotype 'generalized OA,'" consistent with what we found using a different methodology in the present report.

The DIPs and PIPs have long been thought to group together, and bony enlargement of these sites is the basis for a clinical diagnosis of hand OA [29]. MCP involvement is generally thought to be less common than other hand joints and to represent a secondary OA process such as calcium pyrophosphate deposition disease (CPPD) or hemochromatosis. However, as shown by several studies reporting the patterns of hand OA, MCP involvement is not infrequent in radiographic or ultrasonographic OA [6,30], with prevalence estimates ranging from 8% to 36% in Caucasian populations [9,31-33]. MCP rOA has been shown to be more common among African American compared with white women in one study [34]. Our preliminary work on the frequency of hand rOA in the *JoCo OA *showed that although African Americans were much less likely to have DIP or PIP rOA compared with whites, the frequency of MCP rOA was not different by race [35].

Principal components analysis (rather than exploratory factor analysis as described in the current paper) has been used in other studies of radiographic hand OA [20,21]. Marshall et al, reported on a principal components analysis of radiographic variables in the hand [20]. They identified a four-component model where the DIPs (second to fifth digits, right and left), PIPs (second to fifth digits, right and left), and MCP (only included the second and third bilaterally) joints each grouped onto one component and the thumb joints (IP, MCP, CMC, and trapezioscaphoid) onto the fourth. In contrast to the current study, Marshall et al used binary variables in the factor analysis (rOA defined as KL ≥ 2 at each joint), which is not consistent with the assumptions of factor analysis and does not include information about KL severity. In addition, the authors of that study simplified their results to subgroup patients into finger only, thumb only, and thumb and finger together. Although such phenotyping is useful, it does not fully utilize the power of factor analysis to create meaningful composites. Hunter et al, using osteophyte and joint space narrowing scores in addition to KL scores in a principal components analysis of hand OA, chose a 10-factor solution [21]. The DIP joints loaded on a single factor, as did the PIP joints, with other joints (CMC, individual MCPs, thumb IP) loading on separate factors, and these factors were then used in a genetic association study [21]. We found, in common with Marshall et al and Hunter et al, that the MCPs did not load onto the same factor as the IP joints. However, the DIP and PIP joints grouped together in our study along with the CMC, and we did not identify a separate thumb factor, which may be due to our larger sample size or the above-noted differences in methodology.

Although we included data on the hips in our analysis, we found that the hip joints did not load onto any factor. There has long been controversy whether hip OA is part of the "generalized OA" construct, or a separate entity [36-41]. Our attempt to develop a composite measure of multi-joint OA has shown, in agreement with other researchers [6], that the hip is a separate entity, but it remains unclear whether this is a measurement issue or a true difference in the nature of OA at the hip compared with other sites. Arden, et al, in a study assessing different radiographic definitions of hip OA, found that composite measures such as the KL grade had superior construct and predictive validity compared with single radiographic features (such as osteophytes or joint space narrowing alone), and recommended such measures for defining incident hip rOA [42], supporting our use of this grading system. Hip OA is less influenced by obesity in comparison with knee OA [43-45], and may therefore have other unique risk factors that vary by gender and race. For example, among African Americans compared with Caucasians [46], we have reported a higher prevalence of specific radiographic features, such as superior joint space narrowing, previously found to be predictive of progression to hip replacement [47]. Other recently recognized aspects of hip anatomy, such as femoroacetabular impingement and hip shape as described through active shape modeling, may contribute to hip-specific OA risk [48-51] while not reflecting "generalized OA."

Limitations to the current study include the lack of PFJ joint space narrowing data (although PFJ osteophytes were included due to better reproducibility and associations with knee pain [25]), and of radiographs of other joint sites, such as the feet or cervical spine, although we did have radiographic data for many of the joint sites most commonly affected by rOA. Although we used primarily KL grades for the present analysis, use of individual radiographic features (osteophytes and joint space narrowing), could lead to different conclusions and will be the focus of future analyses. Differences in scaling can cause variables to group on a factor due to differences in scale alone, but while we had some radiographic grades that were based on a 0 to 3 scale while others were on a 0 to 4 scale, we did not observe groupings based solely on this difference in scaling. The factor structure presented here has not yet been replicated in an independent population, and should be considered specific to the *JoCo OA *study until replication has been confirmed. The factors we have identified should therefore not be used in other populations without confirmation of a similar structure. We have used only radiographic data in this analysis, although other projects are underway to consider symptoms and other variables of importance to an individual's experience of OA.

## Conclusions

Combination of multiple radiographic variables using composite scores as described allows consideration of the whole body burden of rOA in parsimonious regression models, resulting in reduced dimensionality and increased estimate precision. This methodology provides a way to define more complete phenotypes in individuals with rOA in statistical models, thus improving study of systemic outcomes in this common and debilitating disease.

## Abbreviations

BMI: body mass index; CMC: carpometacarpal joint; DIP: distal interphalangeal joint; DN: disc narrowing; IP: interphalangeal joint; KL: Kellgren-Lawrence; L1/2, etc: lumbosacral spine level; LS: lumbosacral spine; MCP: metacarpophalangeal joint; OA: osteoarthritis; OST: osteophytes; PFJ: patellofemoral joint; PIP: proximal interphalangeal joint; rOA: radiographic osteoarthritis; TFJ: tibiofemoral joint.

## Competing interests

The authors declare that they have no competing interests.

## Authors' contributions

AEN, RFDeV and JMJ were responsible for study conception and design, data acquisition, analysis and interpretation, drafting and critical revision for important content. PGC and VBK were responsible for study conception and design, drafting and critical revision for important content. JBR and TAS were responsible for data acquisition, analysis and interpretation, drafting and critical revision for important content. All authors have read and approved the final manuscript for publication.
